# Clinical Lectures on Diseases of the Eye

**Published:** 1868-04-01

**Authors:** 


### EDITORIAL.

In the course of a few days, those of our subscribers who are in arrears for The Journal, will receive a notification of their indebtedness. They will confer a favor upon us, and save further annoyance, by remitting us promptly on receipt. It is much easier and pleasanter, to both publisher and subscriber, to conduct a publication upon a cash in advance basis, as it is far easier to pay upon the presentation of a bill than to suffer it to drag along. In the next number will be published a list of receipts for subscriptions since January.

Publisher.

### *Clinical Lectures on Diseases of the Eye.*

Dr. E. L. Holmes will deliver two clinical lectures on diseases of the eye, each week during the spring, at the Chicago Charitable Eye and Ear Infirmary.

No more excellent opportunities in the North-west can be secured for the clinical study of the diagnosis and medical and surgical treatment of diseases of the eye, than at the Infirmary. During the past year, 656 patients were treated at this institution. During the entire winter course of lectures, there were in daily attendance at least 36, and for a period of two months, 48 patients.

Certificates of attendance will be given to physicians and students who attend the whole course.

### *Mislaid.*

Some gentleman sent a letter, a few days since, with postal currency to balance, with back numbers of the Journal, for a copy of "*Medical Examinations for Life Insurance.*" The book was wrapped to send him, but in the interim, not more than half an hour, our correspondent's letter, with his address, mysteriously disappeared. It is feared the colored gentleman who does the office fires, etc., is at the bottom of this, as of certain more public difficulties. Will our correspondent write again?

All mistakes at this office will be promptly and most cheerfully rectified.

☞ No more back numbers wanted.

